# CRITICAL SOCIAL GERONTOLOGY AND RURAL AGING

**DOI:** 10.1093/geroni/igz038.1474

**Published:** 2019-11-08

**Authors:** Vanessa Burholt, thomas Scharf

**Affiliations:** 1 Swansea University, Swansea, United Kingdom; 2 Newcastle University, Newcastle, England, United Kingdom

## Abstract

This paper examines the extent to which critical gerontology has raised awareness of the heterogeneity of rural ageing in High Income Countries (HICs) and compare this to our knowledge of the issues that are associated with rural ageing in Low to Middle Income Countries (LMICS). We will draw on Nancy Fraser’s social justice framework to summarize key issues around: (1) Demography (such as globalization, urbanization, counter-urbanization and rural population ageing); (2) Resources (individual material and social resources; community resources such as access to services); (3) Recognition (social status, cultural visibility through social participation and cultural worth through valued social roles); (4) Representation (in social, health and rural development policies; and in private sector and NGO approaches). We argue that an intersectional approach that takes into account location and context (structural/economic/political) alongside other dimensions of oppression and/or privilege can provide a better understanding of the experience of ageing in rural areas.

